# The prognostic significance of the neutrophil-to-lymphocyte ratio and the platelet-to-lymphocyte ratio in giant cell tumor of the extremities

**DOI:** 10.1186/s12885-019-5511-x

**Published:** 2019-04-08

**Authors:** Zhenhao Chen, Guanglei Zhao, Feiyan Chen, Jun Xia, Li Jiang

**Affiliations:** Department of Orthopaedic Surgery, Huashan Hospital, Fudan University, No. 12 Wulumuqi Road(M), Shanghai, 200040 China

**Keywords:** Neutrophil-to-lymphocyte ratio, Platelet-to-lymphocyte ratio, Giant cell tumor, Prognosis

## Abstract

**Background:**

In this study, the influence of the neutrophil-to-lymphocyte ratio (NLR) and the platelet-to-lymphocyte ratio (PLR) on the prognosis of giant cell tumor (GCT) of the extremities were investigated.

**Methods:**

The clinical parameters of 163 patients who were diagnosed with GCT of the extremities between July 2008 and January 2018 were retrospectively analyzed. Optimal cutoff values of NLR and PLR were determined using receiver operating characteristic (ROC) analysis. According to optimal cutoff values, patients were divided into high NLR and low NLR groups or high PLR and low PLR groups. Kaplan-Meier and log-rank methods were used to compare the recurrence-free survival (RFS) between the high and low NLR groups, and between the high and low PLR groups. Univariate analysis was performed to determine the influence of age, gender, neutrophil count, lymphocyte count, platelet count, white blood cell count, tumor size, surgical approach and Campanacci stage on the prognosis of giant cell tumor of bone. The main predictors of RFS were determined by Cox multivariate regression analysis.

**Results:**

The optimal cutoff value of NLR in giant cell tumor of the extremities was 2.32, which was used to classify patients into high and low NLR groups. The optimal cutoff value of PLR was 116.81, and was used to classify patients into high and low PLR groups. Campanacci stage, tumor maximum diameter, alkaline phosphatase, and C-reactive protein (CRP) were significantly associated with the high NLR and PLR. Cox multivariate regression analysis revealed that the Campanacci stage (HR = 3.28, 95% CI: 1.24~8.69) and NLR (HR = 4.18, 95% CI: 1.83~9.57) were independent prognostic factors for giant cell tumor of the extremities.

**Conclusion:**

As a novel inflammatory index, NLR has some predictive power for the prognosis of patients with giant cell tumor of the extremities.

## Background

Giant cell tumor (GCT) is a common primary borderline bone tumor characterized by monocytic-macrophages produced by mononuclear cells and mononuclear spindle-shaped (fibro-osteoblast-like) stromal cells [1, 2]. GCT is prevalent among young people between the ages of 20 and 40 [3]. According to WHO statistics, giant cell tumor of bone accounts for 8.63% of all primary bone tumors [4], and approximately 80% of giant cell tumors of bone are benign with a local recurrence rate of approximately 20–50%. Conventional surgical procedures for GCT include lesion curettage and segmental resection [5–7]. However, lesion curettage has a relatively high local recurrence rate, and segmental resection may result in poor functional recovery and a series of reconstruction-related complications.

Due to the lack of large-scale prospective studies, it is difficult to accurately predict the prognosis of giant cell tumor of bone. Although many studies reported that tumor size, tumor grade and surgical approach influence the prognosis of GCT [8–11], the prognosis of this condition, even with the same grade and surgical procedure, is highly heterogeneous. Therefore, understanding the pathogenesis of giant cell tumor of bone and discovering new prognostic biomarkers would greatly improve its clinical treatment.

Accumulating evidence suggests that tumor progression and prognosis depend not only on tumor characteristics but also on the host inflammatory response and the immunity of the body [12–15]. Approximately 25% of cancers are associated with infection or inflammation which are triggered by different factors [16, 17]. Many cytokines produced during tumor growth, such as interleukins (ILs), vascular endothelial growth factor (VEGF) and tumor necrosis factor (TNF), stimulate the production of granulocytes and platelets [18–21]. These cytokines are also associated with cell proliferation, invasion, migration, angiogenesis and metastasis. Neutrophils release enzymes that remodel the extracellular matrix and produce VEGF, thereby promoting the migration and growth of tumor cells [22]. Activated platelets release large amounts of growth factors that modulate inflammatory responses and promote tumor growth and metastasis [23]. Cellular immunity plays a key role in regulating resistance to tumors. Lymphocytes are the main immune cells of the human body and are important components of specific immune responses to tumors. Lymphocytes regulate the production of cells involved in the tumor-killing process inhibiting tumor progression [24, 25]. Neutrophils inhibit cytotoxic T cells and lymphokine-activated anti-tumor cells thereby causing immune tolerance of tumor antigen, allowing tumors to escape the immune system [26].

Therefore, the ratio of neutrophils to lymphocytes can be used to assess the inflammatory and immune state of the body. This concept can also be applied to the ratio of platelets to lymphocytes. The prognostic value of the ratio of neutrophil-to-lymphocyte (NLR) and the ratio of platelet-to-lymphocyte (PLR) has been evaluated in various tumors, such as gastric cancer, lung cancer, and colorectal cancer, which implies that NLR and PLR have the potential to predict tumor prognosis and response to treatment [27–31]. As a component of the routine blood examinations, NLR and PLR are simple and cost-effective markers of inflammation [31]. However, the potential of inflammatory markers as prognostic indicators for giant cell tumor of bone has not been sufficiently investigated. In this study, we investigated the clinical value of NLR and PLR in the development of GCT of bone by determining the correlation between NLR and PLR of GCT patients with the characteristics of the tumor and the prognosis of the patients.

## Methods

### Patients

We retrospectively reviewed the clinical data of patients diagnosed with giant cell tumor of the extremities at Huashan Hospital, affiliated with Fudan University, between July 2008 and January 2018. The inclusion criteria comprised the following: (1) All patients with histopathological diagnosis of giant cell tumor of the extremities were classified according to the Campanacci method [1]; (2) All patients underwent expanded curettage or extensive resection according to standard surgical procedures; (3) All inflammatory markers were measured before anti-tumor therapy, such as surgery; (4) No blood disease, infection, or fever; (5) No chronic diseases, such as diabetes and chronic obstructive pulmonary disease; (6) All patients were treated for the first time in our hospital. A total of 163 patients with giant cell tumor of the extremities were finally included in the study. The current research was approved by the Huashan Hospital institutional review board (protocol number 2017–338). Informed consent was waived for all participants by the institutional review board as the current study satisfied all of the following requirements for the waiver of informed consent: the research involved a minimal risk to the participants (being a retrospective data analysis of previously collected medical records).

### Clinical parameters

The following clinical parameters were collected for each patient: age, gender, tumor size, surgical approach, tumor stage, alkaline phosphatase (ALP), white blood cell count, percentage of neutrophils, percentage of lymphocytes, platelets, erythrocyte sedimentation rate (ESR) and CRP. ALP, white blood cell count, percentage of neutrophils, percentage of lymphocytes, platelets, ESR and CRP were measured within 3 days prior to surgery as part of the routine preoperative assessments of patients. The NLR and PLR were calculated separately.

### Patient follow-up

Patient follow-up was initiated after surgery. Some patients were followed through outpatient hospital visits, and others were followed through telephone calls. The visiting intervals were 3 months for the first 2 years after surgery, 6 months from the third to the fifth year and 1 year after the fifth year. The deadline for the follow-up was July 2018. Routine follow-ups included physical examination, laboratory examination, and imaging examination, including X-ray, CT, MRI, etc., if necessary. In addition, during the follow-up, functional evaluation of patients was performed according to the Musculoskeletal Tumor Society (MSTS) scoring system [32]. The assessment parameters of the lower limbs included pain, function, emotion, supports, walking and gait. Each score included 5 points, with a total of 30 points. The parameters of the upper limbs included pain, function, emotion, hand positioning, manual dexterity and lifting ability. Each score included 5 points, with a total of 30 points. Since giant cell tumor of bone is a low-invasive tumor, the mortality rate was extremely low, and most of the adverse events lead to local recurrence. Therefore, we selected recurrence-free survival (RFS) as the research index for the prognosis of giant cell tumor of the extremities. The best prognosis was defined as no recurrence until the last follow-up. The recurrence was diagnosed by X-ray, CT or MRI and corroborated by additional histopathological diagnosis. The follow-up period was defined as the interval from the first postoperative day to the diagnosis of recurrence or the end of the follow-up.

### Statistical analysis

The data was sorted using Excel 2007 and analyzed using SPSS 22.0 software (SPSS, Inc., Chicago, IL, USA). The measurement data that obeyed normal distribution were expressed as the means ± standard error (SEM), and those data that disobeyed normal distribution were presented using the median (quartile). The count data were presented as frequency or rate. The optimal cut-off values of NLR and PLR were determined using the receiver operating characteristic (ROC) analysis. Any two groups of data that relatively obeyed normal distribution was analyzed by t-test, and those data that disobeyed normal distribution were analyzed by rank sum test. The chi-squared test was used to compare count data. The survival curves were drawn using the Kaplan-Meier method and compared by the log-rank test. In the analysis of prognostic factors, both univariate analysis and multivariate analysis were performed using Cox regression. The factors were considered to be statistically significant at *p* < 0.05. All confidence intervals (CI) were expressed as 95% confidence level. The absolute neutrophil count was divided by the absolute lymphocyte count to obtain the NLR. The ratio of absolute platelet count to absolute lymphocyte count was calculated as the PLR.

## Results

### Clinical parameters of patients with giant cell tumor of the extremities

Among the 163 patients with giant cell tumor of the extremities, 91 were male (55.83%), and 72 were female (44.17%). The median age was 37 years. According to the Campanacci grading criteria, 86 patients (52.76%) were in stages I and II, and 77 patients (47.24%) were in stage III. Ninety-four patients (57.67%) underwent expanded curettage while 69 patients (42.33%) underwent extensive resection. The median tumor size was 5 cm. The median values of lymphocytes, neutrophils, platelets, ALP, CRP, and ESR were 55.40, 29.50%, 232*10^9^/L, 79 U/L, 8.16 mg/L and 17 mm/h, respectively. The median NLR and PLR were 1.87 and 112.15, respectively.

### The optimal cutoff value of NLR and PLR

**Fig. 1:**
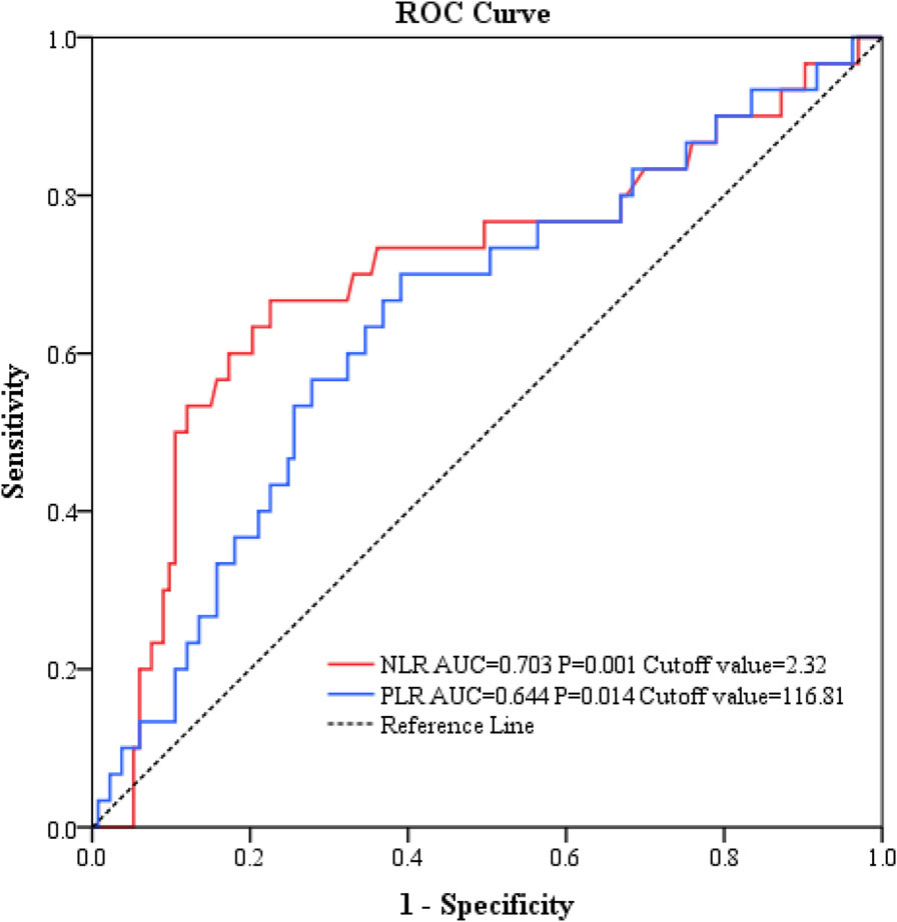
ROC curve for RFS with respect to NLR and PLR in patients with giant cell tumor of the extremities. Cutoff value of NLR, 2.32; Cutoff value of PLR, 116.81. ROC, receiver operating characteristic; RFS, recurrence-free survival

The recurrence outcome of patients with giant cell tumor was used as the endpoint, and Fig. 1 shows the results of ROC analysis of NLR and PLR. The analysis showed that the area under the curve (AUC) corresponding to NLR and PLR were 0.703 (*p* = 0.001) and 0.644 (*p* = 0.014), respectively and were statistically significant. At the highest Youden index, the indicator threshold is the best for distinguishing the predicted target variable [33]. Thus, the optimal cutoff values of NLR and PLR were 2.32 (sensitivity, 66.67%; specificity, 77.44%) and 116.81 (sensitivity, 70.00%; specificity, 60.90%), respectively. The Youden indices of NLR and PLR were 0.441 and 0.309, respectively. Therefore, based on the optimal cutoff values of NLR and PLR, patients were divided into the high NLR group (HNLR ≥2.32) and low NLR group (LNLR < 2.32), as well as the high PLR group (HPLR ≥116.81) and low PLR group (LPLR < 116.81).

### Association of the patients’ clinical parameters with NLR and PLR

The relationship between NLR and PLR with clinical parameters of patients with giant cell tumor of the extremities is shown in Table 1. In NLR groups, the number of patients with stage III tumors in high NLR group was significantly higher than that in low NLR group (*p* < 0.001). The largest tumor diameter (*p* = 0.048), ALP (*p* = 0.047), neutrophil percentage (*p* < 0.001), and CRP (*p* = 0.043) of patients in high NLR group were higher than those in low NLR group. The percentage of lymphocytes in patients of high NLR group (*p* < 0.001) was lower than that in low NLR group. In PLR groups, the number of patients with stage III tumors in high PLR group was significantly higher than that in low PLR group (p < 0.001). The largest tumor diameter (*p* = 0.008), ALP (*p* = 0.008), platelet (*p* < 0.001), and CRP (*p* = 0.005) in high PLR group were higher than those in low PLR group.

**Table 1:** Association of the patients’ clinical parameters with NLR and PLR

clinical parameters	total	NLR		*p*-value	PLR		*p*-value
		LNLR (*n* = 113)	HNLR (*n* = 50)		LPLR (*n* = 90)	HPLR (*n* = 73)	
age (year)	38.49 ± 11.10	38.29 ± 11.18	38.94 ± 11.03	0.732	39.07 ± 10.48	37.78 ± 11.86	0.464
Sex							
Male	91	63	28	0.977	49	42	0.693
Female	72	50	22		41	31	
Companacci stage							
I-II stage	86	70	16	< 0.001	59	27	< 0.001
III stage	77	43	34		31	46	
tumor diameter (cm)	5 (4~7)	5 (4~6)	6 (5~8)	0.048	5 (4~6)	6 (5~8)	0.006
ALP(U/L)	79 (63~122)	76 (62~121)	98 (64~136)	0.047	73 (59~107)	91 (68~124)	0.008
white blood cell (10^9^/L)	6.03 (5.12~7.06)	6.01 (5.13~6.79)	6.20 (5.04~7.30)	0.450	5.95 (5.05~7.06)	6.20 (5.15~7.04)	0.544
neutrophil (%)	55.4 (50.0~61.6)	52.6 (47.8~56.2)	63.6 (61.4~68.4	< 0.001	54.6 (48.8~60.7)	57.1 (50.6~63.2)	0.120
lymphocytes(%)	29.5 (24.2~36.6)	33.8 (29.2~38.9)	22.2 (18.9~24.3)	< 0.001	31.1 (25.6~37.1)	28.2 (23.8~34.2)	0.117
platelet (10^9^/L)	232 (195~272)	231 (191~266)	246 (200~290)	0.159	203 (165~235)	274 (242~314)	< 0.001
ESR (mm/h)	17 (9~34)	16 (9~34)	19 (10~36)	0.439	16 (8~37)	21 (10~34)	0.471
CRP (mg/L)	8.16 (3.77~13.57)	7.64 (3.65~11.93)	12.51 (4.05~23.38)	0.043	6.52 (3.44~11.89)	10.59 (4.39~25.85)	0.005

*ALP*, alkaline phosphatase; *ESR*, erythrocyte sedimentation rate; *CRP,* C-reactive protein; *NLR*, neutrophil-to-lymphocyte ratio; *PLR*, platelet-to-lymphocyte ratio

**Table 2:** Analysis of the factors influencing the postoperative prognosis of patients with giant cell tumor of the extremities

clinical parameters	total	Number of recurrence	Univariate analysis		multivariate analysis	
			HR (95% CI)	*p*-value	Adjusted HR (95% CI)	*p*-value
age (year)	38.49 ± 11.10	30	1.00 (0.96~1.03)	0.769	0.99 (0.95~1.03)	0.541
sex						
Male	91	19	1.00		1.00	
Female	72	11	0.68 (0.32~1.42)	0.302	0.86 (0.39~1.93)	0.717
Companacci stage						
I-II stage	86	6	1.00		1.00	
III stage	77	24	5.95 (2.42~14.63)	< 0.001	3.28 (1.24~8.69)	0.017
surgical method						
expanded curettage	94	17	1.00			
extensive resection	69	13	1.15 (0.56~2.37)	0.708		
tumor diameter (cm)	5 (4~7)	30	1.12 (0.93~1.36)	0.234		
ALP(U/L)	79 (63~122)	30	1.01 (1.00~1.01)	0.026	1.00 (0.99~1.00)	0.284
white blood cell (10^9^/L)	6.03 (5.12~7.06)	30	1.08 (0.84~1.39)	0.539		
ESR (mm/h)	17 (9~34)	30	1.01 (0.99~1.03)	0.232		
CRP (mg/L)	8.16 (3.77~13.57)	30	1.02 (1.01~1.03)	0.004	1.02 (0.99~1.04)	0.199
NLR						
LNLR	113	10	1.00		1.00	
HNLR	50	20	5.90 (2.75~12.67)	< 0.001	4.18 (1.83~9.57)	0.001
PLR						
LPLR	90	9	1.00		1.00	
HPLR	73	21	3.17 (1.45~6.92)	0.004	2.15 (0.90~5.14)	0.084

*HR*, hazard ratio; *CI*, confidence interval; *ALP*, alkaline phosphatase; *ESR*, erythrocyte sedimentation rate; *CRP,* C-reactive protein; *NLR*, neutrophil-to-lymphocyte ratio; *PLR*, platelet-to-lymphocyte ratio; *LNLR*, low NLR; *HNLR*, high NLR; *LPLR*, low PLR; *HPLR*, high PLR

### Prognosis condition of patients with giant cell tumor of the extremities after surgery during follow-ups

**Fig. 2:**
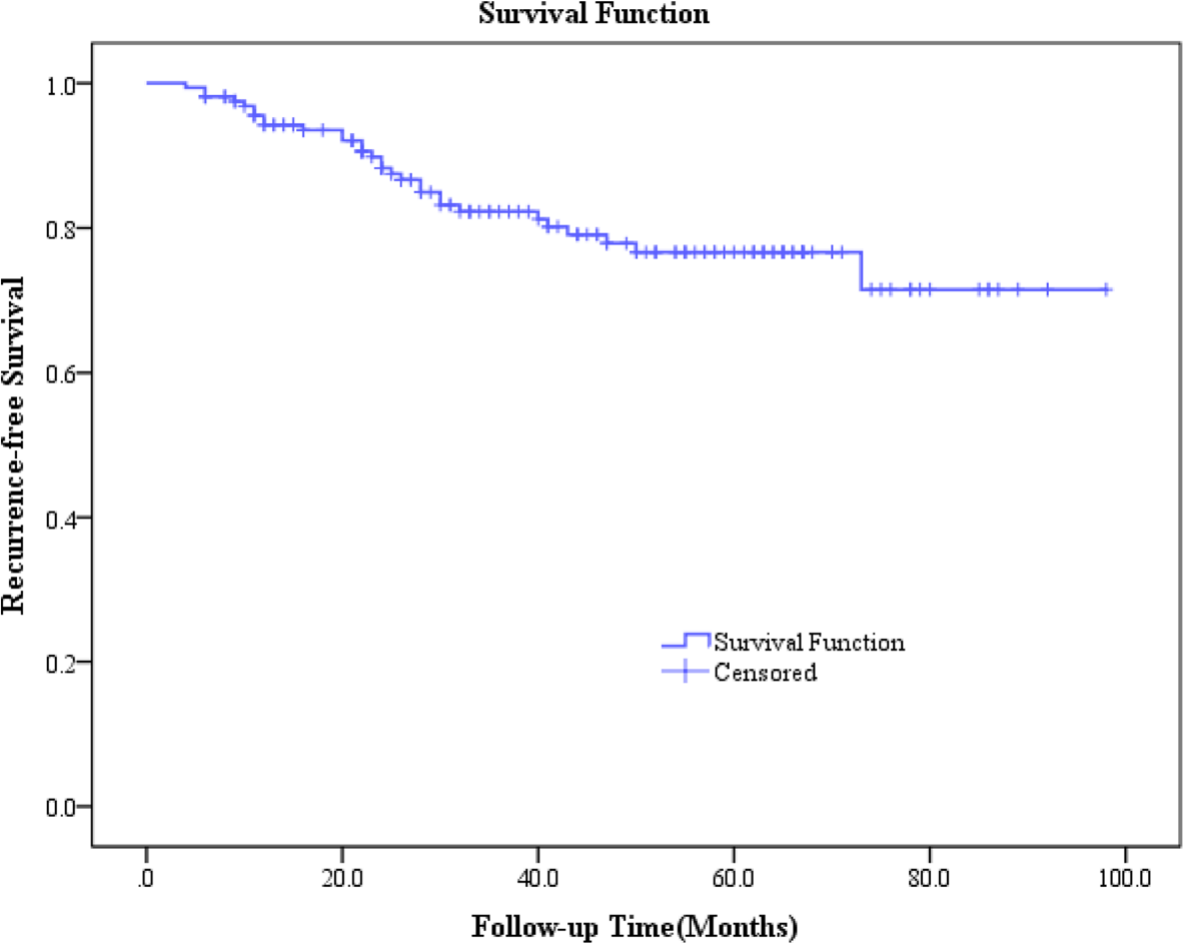
Recurrence-free survival curve of patients with giant cell tumor of the extremities after surgery. The recurrence-free rates of patients at 1 year, 3 years, and 5 years after surgery were 94.27, 82.27, and 76.62%, respectively

The 163 patients with giant cell tumor of the extremities were followed for a maximum of 98 months. The median follow-up time was 37 (22–62) months. During the follow-up, 15 patients were lost to follow-up because the researchers were unable to reach these patients or their families. In addition, recurrence was recorded in 30 patients. The recurrence-free survival curve of patients with giant cell tumor is shown in Fig. 2. According to the analysis, the recurrence-free rates of patients with giant cell tumor at 1 year, 3 years, and 5 years after surgery were 94.27, 82.27, and 76.62%, respectively (Fig. 2).

### Association between NLR and PLR ratios and the survival of patients with giant cell tumor of the extremities

**Fig. 3:**
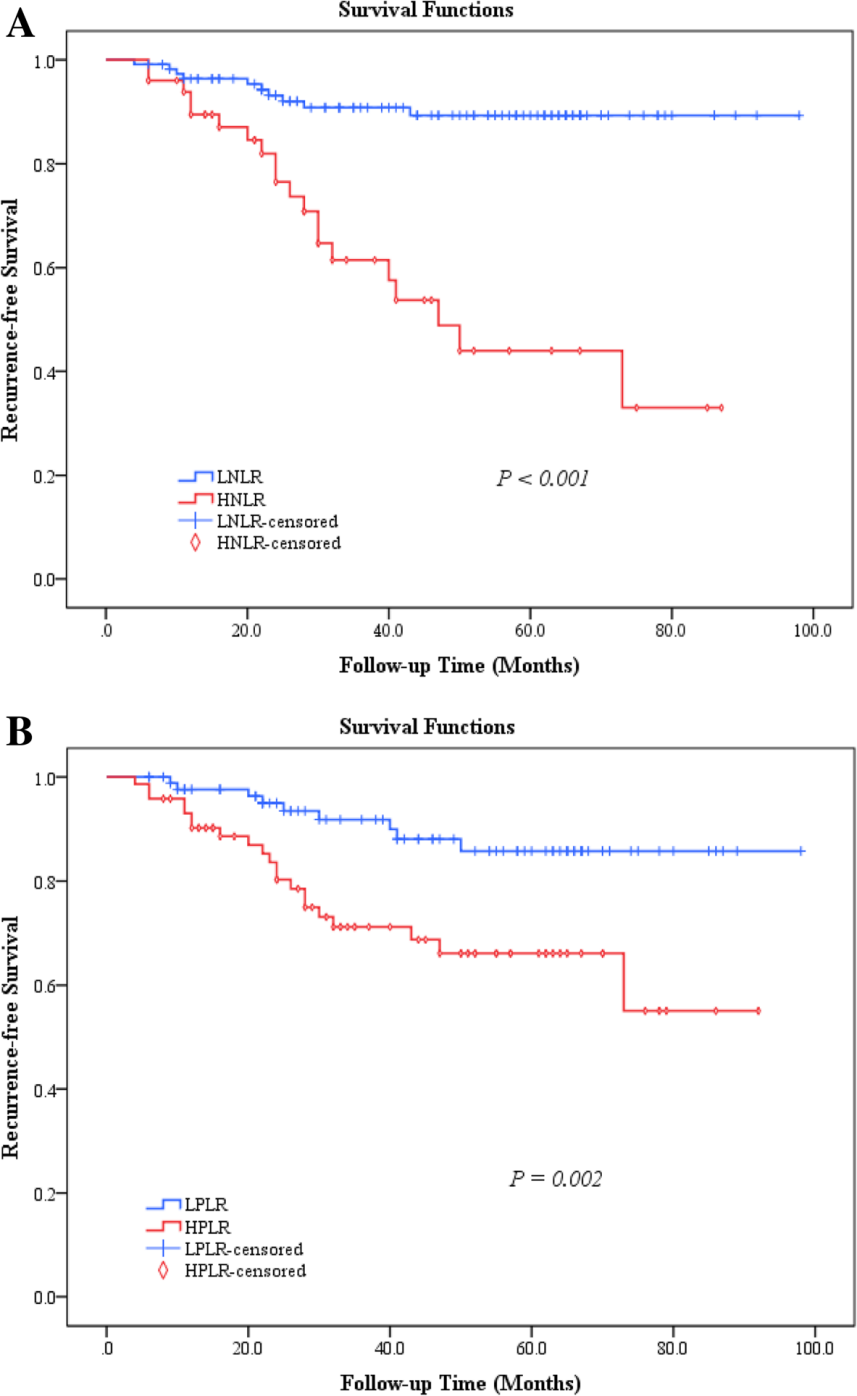
Postoperative recurrence-free survival curve of patients with giant cell tumor in different NLR and PLR groups. **a** The recurrence rate of tumors in patients in low NLR group was lower compared to that in high NLR group. **b** The recurrence rate of tumors in patients of low PLR group was lower than those in high PLR group

Figure 3a shows the recurrence-free survival curve of patients with high NLR and low NLR developed using the Kaplan-Meier method. Comparative analysis showed that patients in the low NLR group had a lower recurrence of tumors compared to those in the high NLR group (χ^2^ = 26.705, *p* < 0.001, log-rank test). The recurrence-free survival curve of patients with high PLR and low PLR is shown in Fig. 3b. Comparative analysis showed that patients in the low PLR group had a lower recurrence rate of tumors than those in the high PLR group (χ^2^ = 9.371, *p* = 0.002, log-rank test).

### Factors influencing the postoperative prognosis of patients with giant cell tumor of the extremities

Cox regression univariate analysis was used to determine the factors that influenced the recurrence rate in Table 2. Campanacci stage, ALP, CRP, the NLR and the PLR were found to be significant determinants of prognostic outcome. These factors were therefore included in the Cox regression to carry out multivariate analysis together with the consideration of age and gender. The results in Table 2 showed that Campanacci stage and NLR were independent factors that influenced the recurrence rate of tumors. Specifically, an advanced clinical stage was a risk factor for recurrence of giant cell tumor (HR = 3.28, 95% CI: 1.24~8.69), and a high NLR (HR = 4.18, 95% CI: 1.83~9.57) was a risk factor for the recurrence of giant cell tumor after surgery.

### The correlation of NLR and PLR with MSTS scores

**Fig. 4:**
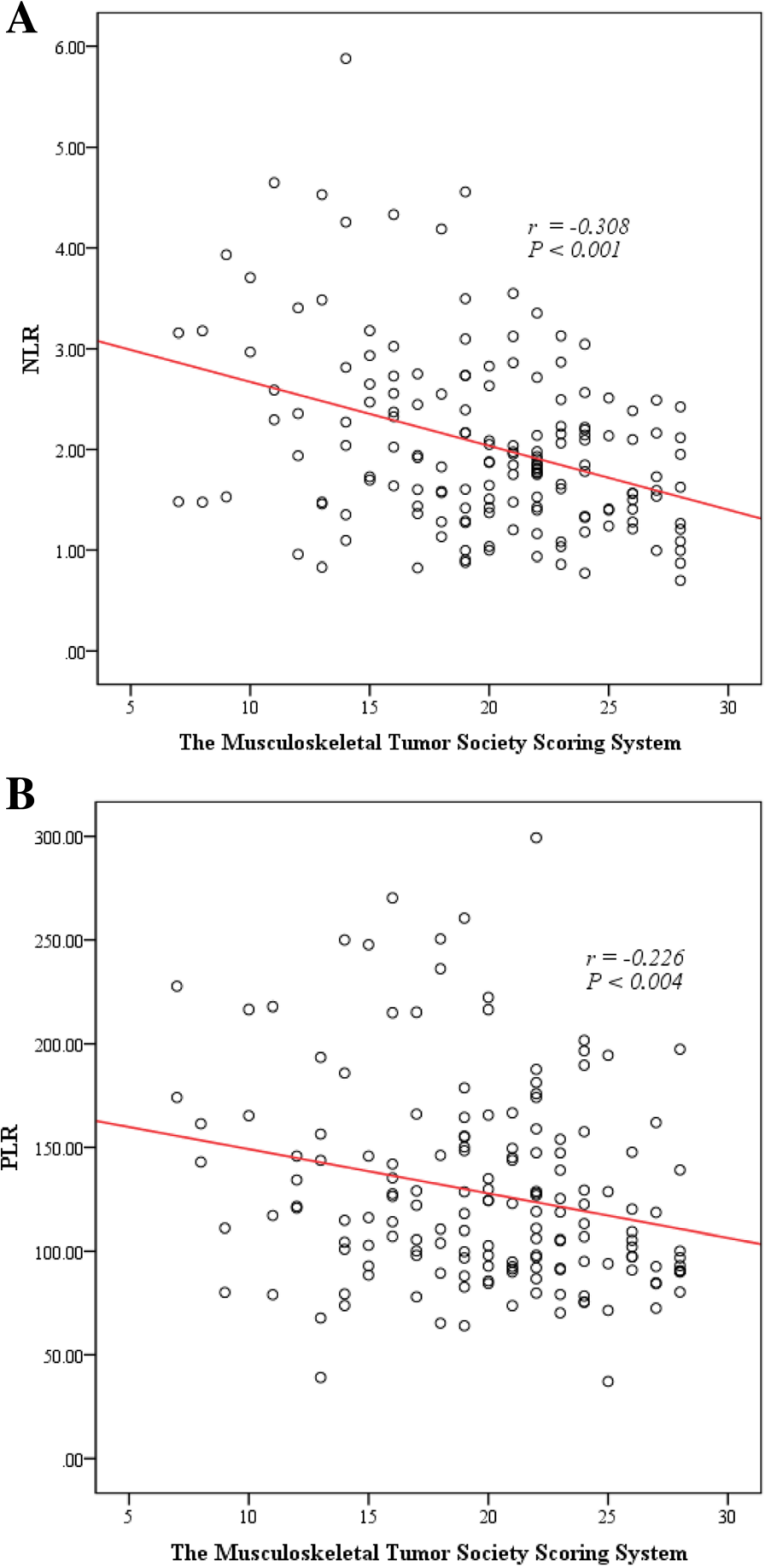
Scatter diagram showing the correlation of NLR and PLR with MSTS scores. **a** Scatter diagram showing a negative correlation between NLR and MSTS (r = − 0.308, *p* < 0.001). **b** Scatter diagram showing a negative correlation between PLR and MSTS (r = − 0.226, *p* = 0.004)

The MSTS scoring system was used to perform functional assessment of patients during the follow-up. The MSTS scoring system has a total of six parameters with 30 points. Correlation scatter plots among NLR, PLR, and the functional scores are shown in Fig. 4a and b. It was found that there was a negative correlation between NLR and MSTS scores. The Spearman correlation coefficient was r = − 0.308, *p* < 0.001; that is, a higher NLR value corresponded to a lower recovery score. Similarly, there was a negative correlation between PLR and MSTS score. The Spearman correlation coefficient was r = − 0.226, *p* = 0.004; that is, a higher PLR value correlated with a lower recovery score.

## Discussion

NLR and PLR have been shown to predict the clinical response to treatment of various solid tumors. Before initiation of chemotherapy, Kim et al. [27] found that NLR could be used as a prognostic factor for metastatic gastric cancer. In addition, the level of NLR after chemotherapy may be used to predict chemotherapy response and prognosis. A review by Lin et al. [34] found that PLR had different predictive values for postoperative liver cancer of different grades and had a positive predictive effect on the prognosis of C-stage cancer based on Barcelona staging. The relationship between inflammatory markers and the prognosis of giant cell tumor of bone has not been sufficiently studied. This study showed that both NLR and PLR influenced the recurrence of giant cell tumor of the extremities based on univariate analysis. Further analysis revealed that a high preoperative NLR was an independent risk factor for postoperative recurrence of giant cell tumor of the extremities, but a high PLR was not, which is consistent with the results of He et al. [35]. In their study, these authors stated that NLR is superior to PLR in predicting poor prognosis of metastatic colorectal cancer.

Yildirim et al. [31] explored the diagnostic value of NLR in malignant tumors and proposed that the level of NLR could be used to distinguish benign and malignant ovarian masses. It has been shown that malignant tumors, but not benign tumors, are often caused by severe inflammatory reactions and lymphocyte suppressive responses acting at the same site. The giant cell tumor of bone is defined as “invasive, potentially malignant lesions” by the World Health Organization [36]. This study confirmed that the giant cell tumor of bone is invasive, and the index of inflammatory response was stronger in recurrent giant cell tumor of bone than in non-recurrent type. Survival analysis showed that the RFS of the low NLR and low PLR groups of giant cell tumor of the extremities were higher than those of the high NLR and high PLR groups. The likelihood of recurrence and invasiveness of giant cell tumor of bone was higher in patients with high NLR and high PLR values.

In addition, studies have shown that age, gender and tumor size do not influence the recurrence rate [37–39]. Our results are consistent with the previous findings. In addition to other physical and chemical treatments, the giant cell tumor of bone is mainly treated by surgical treatment. Other treatments include grinding, argon, liquid nitrogen or phenol to inactivate residual tumor cells. The current surgical methods are effective in reducing the recurrence rate of giant cell tumor of bone, including lesion curettage for stage I and II giant cell tumor and segmental resection for stage III giant cell tumor. However, the impact of both surgical methods on the prognosis of giant cell tumor of bone remains unclear, and the results from previous studies are not conclusive [6]. Li et al. [40] and Niu et al. [3] compared the effects of intralesional curettage, expanded curettage and extensive resection on the prognosis of giant cell tumor of bone. They found that the type of surgical approach was an independent risk factor for recurrence of giant cell tumor of the extremities. Compared with intralesional curettage, the expanded curettage approach combined with bone cement, bone graft, or a combination of both and the extensive resection approach with or without reconstruction had a lower recurrence rate. In this study, univariate analysis showed that the surgical approach did not influence the recurrence of giant cell tumor of bone, which is inconsistent with the results of Li et al. and Niu et al. It is possible that, in our study, all patients underwent expanded curettage or extensive resection, which are both relatively aggressive surgical approaches, thus the surgical approaches might have similar effects on the prognosis.

Currently, data regarding the effective functional scores after treatment of giant cell tumor of bone are scarce. According to some studies, the functional scores obtained from a combination of curettage and other treatments were better than those obtained from expanded resection as there was a lower incidence of non-neoplastic complications. However, this difference was not statistically significant [41]. Correlation analysis of the postoperative MSTS scores in this report showed that patients with high NLR and PLR values had lower postoperative MSTS scores, which is in line with the results reported by Errani et al. [38]. This finding is likely due to the fact that there were more patients with stage III tumors in the high NLR and PLR groups. Patients in these groups underwent extensive resection or functional reconstruction, which would cause more postoperative complications, thereby affecting the patient’s functional recovery. Meanwhile, the high NLR and PLR groups had higher RFS which will lead to worse functional recovery.

The clinical landscape of giant cell tumor of bone is not well-understood. Several studies have reported inconsistent results about the impact of age, sex, tumor size, or clinical grade on the prognosis of GCT patients [10, 42, 43]. Interestingly, even the histological features of GCT of bone are not considered to be determinants of whether the tumor relapses according to many studies [44]. This study demonstrates, for the first time, that NLR can predict the prognosis of giant cell tumor of the extremities. This finding provides insights related to the pathogenesis and treatment of giant cell tumor of bone. GCT is a true neoplastic process originating from undifferentiated mesenchymal cells of the bone marrow [4]. Neutrophils, ordinarily, will not be released from the bone marrow until mature. However, in the context of inflammation, they are produced following the release of cytokines and chemokines, such as IL-6, TNF and myeloid growth factors. These inflammatory mediators enhance the invasion, tumor growth, angiogenesis and metastasis of tumor, aid tumor cells to evade immune surveillance, and induce resistance to cytotoxic drugs [12]. Elsewhere, it was reported that platelets and neutrophils can promote adhesion and seeding of distant organ sites by inducing the secretion of VEGF [23, 24]. On the contrary, lymphocytes are basic components of the adaptive and innate immune system and simultaneously involved in immune-surveillance and immune-editing. Thus, decreased lymphocyte count and function will impair immune surveillance and defense [45]. Inflammation induces changes in the cancer microenvironment that favor tumor progression. The roles of platelets, lymphocytes and neutrophils in relation to tumor support the conclusion of this study, that is, NLR and PLR have prognostic significance in giant cell tumor of the extremities. For patients with giant cell tumor of the extremities with high NLR values, whatever the tumor stages are, adjuvant treatment and medication such as Denosumab (human monoclonal antibody of RANKL), combined with surgical resection are recommended to reduce the recurrence rate and improve functional recovery. The expanded curettage approach combined with bone cement implantation is highly recommended because it not only reduces the long-term recurrence rate but also ensures better limb function [42].

However, there are some limitations to this study. First, this study was conducted retrospectively with a small number of patients. Second, some recent patients were included in order to increase the number of samples. Finally, patients with the same tumor were not given identical treatments, which may influence the interpretation of outcomes. Therefore, further large-sample studies are needed to confirm the relationship between inflammatory biomarkers and the prognosis of patients with giant cell tumor of bone.

## Conclusion

Here, we report for the first time that NLR and PLR are associated with postoperative recurrence of giant cell tumor of the extremities. As an independent risk factor for giant cell tumor of bone, NLR can predict the treatment outcomes of this tumor. Patients with giant cell tumor of bone with low NLR values can be given routine treatments, while those with high NLR values can be treated with more aggressive surgical methods combined with adjuvant therapy. As a component of routine blood tests, NLR is easy to measure and convenient to apply in clinical practice. In the future, randomized prospective studies with larger sample sizes are needed to confirm the relationship between inflammation markers and giant cell tumor of bone. Further mechanistic studies will be valuable to reveal the effects of inflammatory factors on the occurrence and development of tumors.

## Abbreviations

ALP: Alkaline phosphatase
AUC: Area under the curve
CI: Confidence intervals
ESR: Erythrocyte sedimentation rate
GCT: Giant cell tumor
HR: Hazard risk
ILs: Interleukins
MSTS: Musculoskeletal Tumor Society
NLR: Neutrophil-to-lymphocyte ratio;
PLR: Platelet-to-lymphocyte ratio
RFS: Recurrence-free survival
ROC: Receiver operating characteristic
TNF: Tumor necrosis factor
VEGF: Vascular endothelial growth factor
